# Face size biases emotion judgment through eye movement

**DOI:** 10.1038/s41598-017-18741-9

**Published:** 2018-01-10

**Authors:** Shuo Wang

**Affiliations:** 1 Department of Chemical and Biomedical Engineering, West Virginia University, Morgantown, WV 26506 USA; 2 Blanchette Rockefeller Neurosciences Institute, West Virginia University, Morgantown, WV 26506 USA

## Abstract

Faces are the most commonly used stimuli to study emotions. Researchers often manipulate the emotion contents and facial features to study emotion judgment, but rarely manipulate low-level stimulus features such as face sizes. Here, I investigated whether a mere difference in face size would cause differences in emotion judgment. Subjects discriminated emotions in fear-happy morphed faces. When subjects viewed larger faces, they had an increased judgment of fear and showed a higher specificity in emotion judgment, compared to when they viewed smaller faces. Concurrent high-resolution eye tracking further provided mechanistic insights: subjects had more fixations onto the eyes when they viewed larger faces whereas they had a wider dispersion of fixations when they viewed smaller faces. The difference in eye movement was present across fixations in serial order but independent of morph level, ambiguity level, or behavioral judgment. Together, this study not only suggested a link between emotion judgment and eye movement, but also showed importance of equalizing stimulus sizes when comparing emotion judgments.

## Introduction

Faces are among the most commonly perceived visual stimuli and play a key role in social communication. People often form judgments of others based purely on facial features and trait evaluations from faces can predict important social outcomes. For example, inferences of competence based solely on facial appearance predict the outcomes of elections^1^ and facial features can influence sentencing decisions^2^. On the other hand, faces are socially salient stimuli and people preferentially attend to faces^3^. For example, people detect faces faster than inanimate objects (e.g., plants and artifacts) in the change detection task^4^ and people orient more and faster to faces in natural scenes^5^. The way to look at faces has both a developmental^6^ and genetic^7^ root, and it often serves as a biomarker for autism, which shows atypical attention to faces^5,8^.

Humans have a dedicated and distributed network of brain regions to process faces. Intracranial field potential studies in neurosurgical patients^9^ and functional imaging studies^10^ have both provided evidence that cortical areas in the lateral parts of the inferior occipital gyrus, fusiform gyrus, and superior temporal gyrus are associated with face processing (see^11^ for a review). In particular, faces signal important information through expressions of emotions, which in turn provide a strong motivating influence on how the environment is perceived^12^. A large number of brain regions participate in recognizing emotions from facial expressions, including the occipitotemporal cortices, amygdala, orbitofrontal cortex, basal ganglia, and right parietal cortices (see^13^ for a review), among which the amygdala plays a key role in processing facial emotions: the human amygdala encodes not only fear emotion^14,15^ and emotions in general^16^, but also subjective judgment of facial emotions^17^ and categorical ambiguity of emotions^15^. A recent proposal argues that emotion should be understood in terms of large-scale network interactions spanning the entire neuro-axis^18^.

Perception of facial expressions is closely related to eye movement. For example, more fixations are directed to the eye region when people view fearful faces whereas relatively more fixations are directed to the mouth region when people view happy faces^19^. Also, eyes contain more information for fearful faces but the mouth contains more information for happy faces^20^. Human neuroimaging studies have shown that amygdala activity is specifically enhanced for fearful faces and saccades to the eyes^21^, and monkey physiological studies using dynamic social videos with various facial expressions have revealed a subset of neurons in the amygdala that respond selectively to fixations on the eyes of other monkeys and to eye contact^22^. A recent computational framework with novel spatiotemporal analyses of eye movements has provided theoretical insights and empirical evidence for the computational mechanisms underlying perception of facial expressions^23^. This framework has also revealed culture-specific decoding strategies of facial expressions, arguing against the universality of human facial expressions of emotion^24^.

Most studies up to date focus on the diagnostic facial features for emotion judgment (e.g.,^20^) and investigate neural correlates of facial expressions by manipulating the emotion contents (e.g.,^25^). However, it remains unclear whether a simple low-level feature, face size, will influence emotion judgment. In this study, I investigated whether a mere difference in face size would cause different emotion judgments. I employed an emotion judgment task with fear-happy morphed faces. Indeed, when subjects viewed larger faces, they not only had a lower threshold to detect fear on the face, but also showed a higher specificity in emotion judgment. However, subjects showed a similar confidence judgment between face sizes. Concurrent eye tracking further provided insights into the underlying mechanism: more fixations were directed to the eyes when people viewed larger faces whereas there was a wider spatial dispersion of fixations when people viewed smaller faces. This difference was present across fixations in serial order, but independent of the morph level, ambiguity level, and behavioral judgment. Together, this study not only suggested a link between emotion judgment and eye movement, but also showed importance of choosing stimulus size to study emotion judgment.

## Results

### Emotion judgment

**Figure 1:**
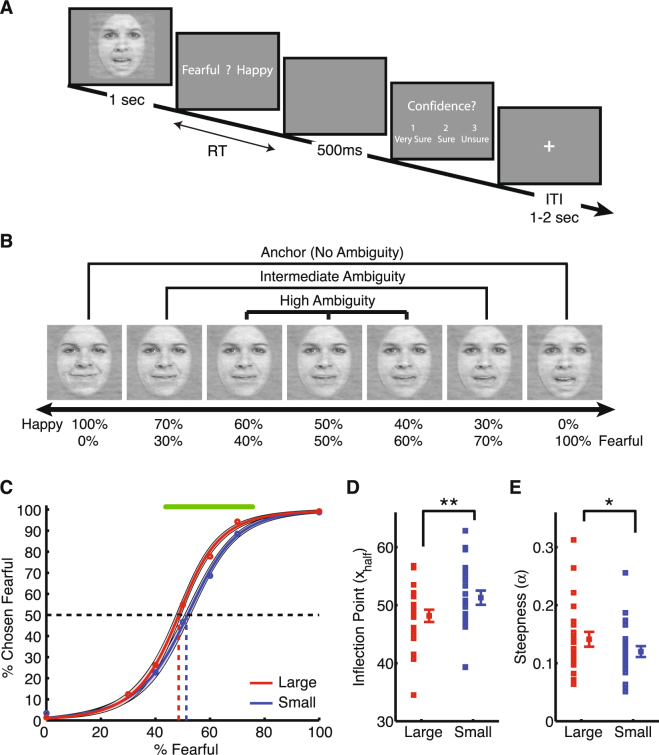
Emotion judgment. **(A)** Task. Subjects viewed a face for 1 second and reported their emotion judgment (fearful or happy). Following emotion judgment, subjects also reported their confidence in emotion judgment (‘1’ for ‘very sure’, ‘2’ for ‘sure’ or ‘3’ for ‘unsure’). **(B)** Example faces from a female face model. Face stimuli were constructed from^44^. **(C)** Psychometric curves for large vs. small faces. Shaded area denotes one SEM across subjects. The top green bar illustrates the points with significant difference between large vs. small faces (paired two-tailed t-test, P < 0.05, FDR corrected). **(D)** Index for emotion judgment bias (*x*_*half*_). **(E)** Index for emotion judgment sensitivity and specificity (*α*). Asterisks indicate significant difference using two-tailed paired t-test. *P < 0.05, and **P < 0.01.

Subjects performed an emotion judgment task (Fig. 1A) with “anchor” (unambiguously happy or unambiguously fearful) and morphed faces (Fig. 1B). Psychometric curves were fitted for each subject (Eq. 1). The proportion of trials identified as fearful monotonically increased as a function of fearful level in the face (Fig. 1C). Two metrics from the fitted psychometric curves were used to compare emotion judgment. First, *x*_*half*_, the midpoint of the curve with equal proportions of fearful and happy judgment, shows emotion judgment bias. I found that subjects had a significantly smaller *x*_*half*_ for large faces compared to small faces (Fig. 1D; large: 48.2 ± 5.09 (mean ± SD), small: 51.3 ± 5.90; paired two-tailed t-test: t(22) = 3.65, P = 0.0014, effect size in Hedges’ g (standardized mean difference): g = 0.56), suggesting that they were more likely to judge faces as fearful when viewing large faces. Second, *α*, the steepness of the psychometric curve, shows emotion judgment sensitivity and specificity. I found that subjects had a greater *α* for large faces (Fig. 1E; large: 0.14 ± 0.061, small: 0.12 ± 0.045; t(22) = 2.29, P = 0.032, g = 0.39), showing that they had steeper psychometric curves when viewing large faces, which in turn suggested that subjects were more sensitive and specific in their emotion judgment when they viewed large faces.

Together, my results show that different face sizes led to not only different thresholds for judging emotions, but also different sensitivity and specificity of emotion judgment.

### Confidence judgment

**Figure 2:**
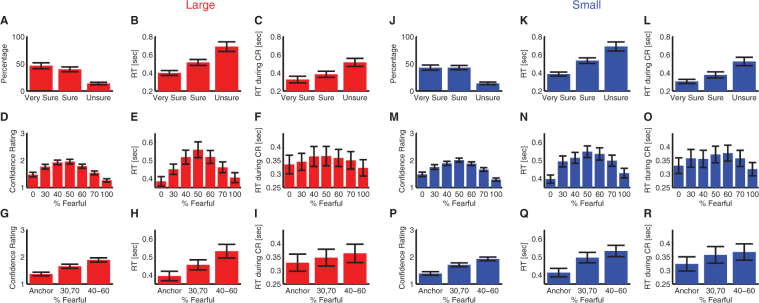
Confidence judgment. **(A**–**I)** Large faces. **(J**–**R)** Small faces. **(A**,**J)** Percentage of trials reporting each level of confidence. **(B**,**K)** RT of emotion judgment for each confidence level. **(C**,**L)** RT of confidence judgment for each confidence level. **(D**,**M)** Confidence rating for each morph level. **(E**,**N)** RT of emotion judgment for each morph level. **(F**,**O)** RT of confidence judgment for each morph level. **(G**,**P)** Confidence rating for each ambiguity level. **(H**,**Q)** RT of emotion judgment for each ambiguity level. **(I**,**R)** RT of confidence judgment for each ambiguity level. Error bars denote ±SEM across subjects.

Besides emotion judgment, subjects also provided confidence judgment in their decisions (Fig. 1A). There were three levels of confidence: ‘Very Sure’, ‘Sure’, and ‘Unsure’. First, I found that subjects reported high confidence more often than low confidence (Fig. 2A,J; one-way repeated-measure ANOVA of confidence levels; large: F(2,46) = 11.2, P = 1.07 × 10^−4^, η^2^ = 0.33; small: F(2,46) = 12.9, P = 3.57 × 10^−5^, η^2^ = 0.36). They judged emotions faster (Fig. 2B,K; large: F(2,43) = 19.6, P = 2.40 × 10^−7^, η^2^ = 0.25; small: F(2,45) = 33.5, P = 1.43 × 10^−10^, η^2^ = 0.31) and reported confidence faster (Fig. 2C,L; large: F(2,43) = 7.80, P = 9.22 × 10^−4^, η^2^ = 0.12; small: F(2,45) = 18.2, P = 6.11 × 10^−7^, η^2^ = 0.19) when they had higher confidence. However, this was similarly the case for both large and small faces (two-way repeated-measure ANOVA of face size X confidence level; main effect of face size: all Ps > 0.88; main effect of confidence level: all Ps < 4.91 × 10^−9^) and there were no interactions between face sizes and confidence levels (all Ps > 0.77).

Second, when I analyzed confidence judgment as a function of morph level, I found that subjects reported higher confidence for anchor faces but lower confidence for ambiguous faces (Fig. 2D,M; one-way repeated-measure ANOVA of morph levels; large: F(6,138) = 42.0, P = 7.54 × 10^−29^, η^2^ = 0.28; small: F(6,138) = 37.2, P = 1.37 × 10^−26^, η^2^ = 0.30). I found a similar relationship not only for reaction time (RT) of emotion judgment (Fig. 2E,N; large: F(6,138) = 19.0, P = 4.93 × 10^−16^, η^2^ = 0.12; small: F(6,138) = 19.0, P = 4.64 × 10^−16^, η^2^ = 0.11), but also RT of confidence judgment (Fig. 2F,O; large: F(6,138) = 2.17, P = 0.050, η^2^ = 0.0086; small: F(6,138) = 6.33, P = 6.68 × 10^−6^, η^2^ = 0.019). However, large faces and small faces had a similar pattern of results (two-way repeated-measure ANOVA of face size X morph level; main effect of face size: all Ps > 0.68; main effect of morph level for explicit confidence rating: P = 1.40 × 10^−56^, RT of emotion judgment: P = 9.01 × 10^−33^, and RT of confidence judgment: P = 6.99 × 10^−7^; interactions: all Ps > 0.41).

Third, when I analyzed confidence judgment as a function of ambiguity level (Fig. 1B,I found that subjects reported higher confidence for anchor faces but lower confidence for ambiguous faces (Fig. 2G,P; one-way repeated-measure ANOVA of ambiguity levels; large: F(2,46) = 72.6, P = 5.82 × 10^−15^, η^2^ = 0.26; small: F(2,46) = 82.8, P = 5.67 × 10^−16^, η^2^ = 0.31). I found a similar relationship for both RT of emotion judgment (Fig. 2H,Q; large: F(2,46) = 31.7, P = 2.25 × 10^−9^, η^2^ = 0.12; small: F(2,46) = 38.5, P = 1.51 × 10^−10^, η^2^ = 0.12), and RT of confidence judgment (Fig. 2I,R; large: F(2,46) = 3.71, P = 0.032, η^2^ = 0.0082; small: F(2,46) = 9.67, P = 3.12 × 10^−4^, η^2^ = 0.018). However, again, large faces had a similar pattern of results as small faces (two-way repeated-measure ANOVA of face size X ambiguity level; main effect of face size: all Ps > 0.62; main effect of ambiguity level for explicit confidence rating: P = 3.45 × 10^−30^, RT of emotion judgment: P = 1.04 × 10^−18^, and RT of confidence judgment: P = 2.64 × 10^−5^; interactions: all Ps > 0.22).

Together, I found very similar patterns of confidence judgment between large vs. small faces, suggesting that face size did not influence confidence judgment.

### Different face sizes led to different spatial distributions of fixations

**Figure 3:**
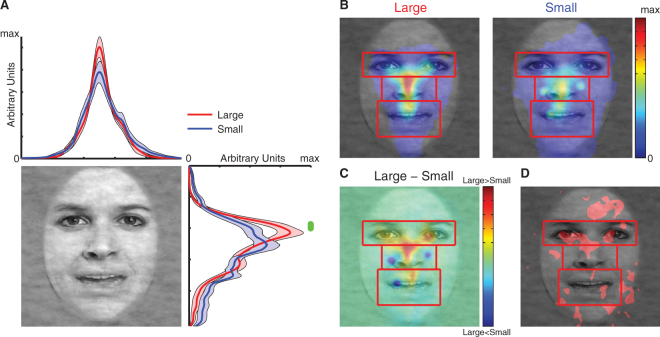
Fixation density comparisons between large vs. small faces. **(A)** 2D probability distribution of fixation densities. Shaded area denotes one SEM across subjects. The green bar illustrates the points with significant difference between large vs. small faces (paired two-tailed t-test, P < 0.05, FDR corrected). **(B)** Fixation density probability maps for large vs. small faces. A common scale (in arbitrary units) is used for both plots. Red rectangles denote the ROIs used for analysis (not shown to subjects). **(C)** A difference map of fixation density. Red: large > small. Blue: small > large. **(D)** A statistical map. Significant differences in fixation density between face sizes are shown in red (two-tailed paired t-test at each pixel, P < 0.05 uncorrected). Face images were constructed from^44^.

Could the difference in emotion judgment be attributed to difference in eye movement? To investigate this question, I next analyzed the spatial distribution of fixations. For each subject, I collapsed fixations from all trials during the 1 s stimulus period. I found that when subjects viewed both large and small faces, the fixation density distribution was symmetric along the horizontal dimension. However, I found a significant difference along the vertical dimension: the fixation density distribution for large faces shifted up towards the eyes, whereas the distribution for small faces remained around the center on the nose (Fig. 3A). Direct comparison at each pixel confirmed the difference along the vertical dimension but not the horizontal dimension (two-tailed paired t-test; green bar in Fig. 3A). Furthermore, fixation density maps (Fig. 3B) showed that large faces led to a narrower dispersion of fixations whereas small faces led to a wider dispersion. This was expected due to the smaller visual angle subtended by small faces—a fixation from the stimulus center with the same distance could travel farther on the stimulus. However, interestingly, when subjects viewed large faces, they not only had more fixations around the stimulus center, but also had more fixations towards the eyes (Fig. 3C). Direct comparisons with individual density maps (two-tailed paired t-test at each pixel, uncorrected with P < 0.05; Fig. 3D) further confirmed such upward shift of fixations. It is worth noting that this finding is consistent with the behavioral results (i.e., increased fearful judgment with large faces): previous literature has shown that people fixate on eyes^19^ and utilize eye information^20^ for fear judgment.

It is worth noting that valid recording durations per trial were similar between large and small faces (large: 920 ± 64.7 ms, small: 934 ± 46.4 ms; two-tailed paired t-test: t(23) = 1.23, P = 0.23, g = 0.25), suggesting that my results could not be attributed to different qualities in eye tracking. Moreover, subjects had more fixations per trial for large faces (3.48 ± 0.70) compared to small faces (2.77 ± 0.73; t(23) = 6.64, P = 8.87 × 10^−7^, g = 0.98), suggesting that subjects had more eye movements when they viewed large faces.

Together, I found that different face sizes resulted in different patterns of eye movement, which in turn led to different emotion judgments.

### Region of interest (ROI) analysis showed that subjects had more fixations onto eyes when they viewed large faces

**Figure 4:**
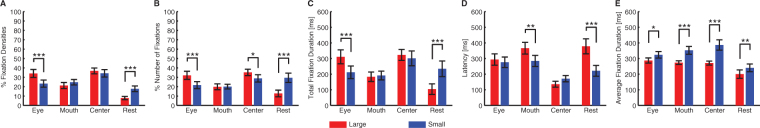
ROI analysis of fixation properties. **(A)** Fixation density. **(B)** Fixation number. **(C)** Total fixation duration. **(D)** Latency of the first fixation onto each ROI. **(E)** Mean fixation duration. Error bars denote ± SEM across subjects. Asterisks indicate significant difference using two-tailed paired t-test. *P < 0.05, **P < 0.01, and ***P < 0.001.

To further characterize the relationship between fixation properties and facial features, I conducted a region of interest (ROI) analysis. First, I analyzed fixation densities in each ROI (Fig. 4A; two-way repeated-measure ANOVA of face size X ROI type; main effect of face size: F(1,138) = 0.31, P = 0.58; main effect of ROI type: F(3,138) = 12.5, P = 2.74 × 10^−7^; interaction: F(3,138) = 2.64, P = 0.052). Confirming the above fixation distribution finding, subjects had a greater tendency to fixate the eyes when they viewed large faces compared to small faces (large: 34.1 ± 20.8% (mean ± SD), small: 23.3 ± 18.5%; two-tailed paired t-test: t(23) = 3.90, P = 7.27 × 10^−4^, g = 0.54), but subjects had more fixations outside ROIs when they viewed small faces (large: 7.88 ± 8.48%, small: 17.7 ± 14.7%; t(23) = 3.83, P = 8.62 × 10^−4^, g = 0.81). No significant difference was found for the mouth nor center ROI (both Ps > 0.05).

I next computed the percentage of the number of fixations in each ROI (Fig. 4B; two-way repeated-measure ANOVA of face size X ROI type; main effect of face size: F(1,138) = 1.80, P = 0.19; main effect of ROI type: F(3,138) = 3.28, P = 0.023; interaction: F(3,138) = 3.78, P = 0.012), which was similar to the fixation density results: large faces attracted more fixations to the eye ROI (large: 32.1 ± 21.2%, small: 21.6 ± 18.4%; t(23) = 4.39, P = 2.14 × 10^−4^, g = 0.52) and center ROI (large: 35.2 ± 16.8%, small: 28.8 ± 19.1%; t(23) = 2.22, P = 0.037, g = 0.35), whereas small faces featured more fixations outside ROIs (large: 12.8 ± 17.3%, small: 29.5 ± 24.5%; t(23) = 5.40, P = 1.76 × 10^−5^, g = 0.77). Similar results were also derived for the total fixation duration in each ROI (Fig. 4C; two-way repeated-measure ANOVA of face size X ROI type; main effect of face size: F(1,138) = 1.20, P = 0.28; main effect of ROI type: F(3,138) = 4.27, P = 0.0065; interaction: F(3,138) = 2.20, P = 0.091): the total fixation duration in the eye ROI was longer for large faces (large: 311 ± 219 ms, small: 212 ± 198 ms; t(23) = 3.82, P = 8.81 × 10^−4^, g = 0.47) whereas it was longer outside ROIs for small faces (large: 104 ± 166 ms, small: 234 ± 245 ms; t(23) = 4.19, P = 3.48 × 10^−4^, g = 0.61). No significant difference was found for the mouth nor center ROI (both Ps > 0.05).

Interestingly, when viewing small faces (Fig. 4D; two-way repeated-measure ANOVA of face size X ROI type; main effect of face size: F(1,137) = 6.59, P = 0.014; main effect of ROI type: F(3,137) = 9.50, P = 9.53 × 10^−6^; interaction: F(3,137) = 2.75, P = 0.045), subjects oriented to the mouth (large: 366 ± 184 ms, small: 285 ± 174 ms; t(23) = 3.05, P = 0.0057, g = 0.45) and outside ROIs (large: 378 ± 235 ms, small: 221 ± 169; t(23) = 4.66, P = 1.09 × 10^−4^, g = 0.75) faster, likely due to shorter distances between facial features; however, this was not the case for eyes (large: 294 ± 177 ms, small: 276 ± 161 ms, t(22) = 0.99, P = 0.33, g = 0.10). Lastly, large faces featured shorter mean fixation duration in all ROIs (Fig. 4E; two-way repeated-measure ANOVA of face size X ROI type; main effect of face size: F(1,137) = 7.96, P = 0.0071; main effect of ROI type: F(3,137) = 15.7, P = 6.46 × 10^−9^; interaction: F(3,137) = 2.38, P = 0.072; eye: large: 287 ± 81.3 ms, small: 324 ± 99.8 ms; t(23) = 2.17, P = 0.042, g = 0.40; mouth: large: 274 ± 58.8 ms, small: 352 ± 123 ms; t(23) = 3.92, P = 6.80 × 10^−4^, g = 0.81; center: large: 271 ± 62.4 ms, small: 387 ± 162 ms; t(23) = 4.09, P = 4.45 × 10^−4^, g = 0.93; outside ROIs: large: 200 ± 132 ms, small: 240 ± 121 ms; t(23) = 3.23, P = 0.0038, g = 0.31).

In conclusion, ROI analysis showed that subjects had more fixations onto eyes when they viewed large faces. However, small faces featured more fixations outside ROIs as well as longer fixations.

### ROI analysis by fixation serial order

**Figure 5:**
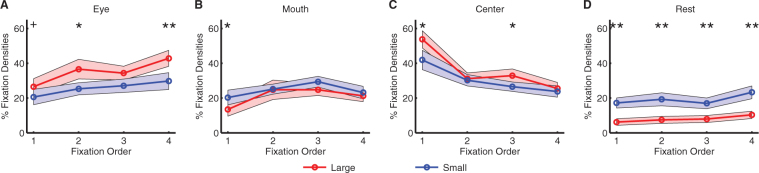
Fixation density as a function of fixation serial order. **(A)** Eye. **(B)** Mouth. **(C)** Center. **(D)** Area not in any of the ROI. Shaded area denotes one SEM across subjects. Asterisks indicate significant difference using two-tailed paired t-test (uncorrected). +P < 0.1, *P < 0.05, and **P < 0.01.

The results shown above used all fixations from the trial. However, were there differences at particular individual fixations? To answer this question, I next analyzed fixation densities in serial order (Fig. 5; three-way repeated-measure ANOVA of face size X ROI type X fixation order; main effect of ROI type: F(3,678) = 41.3, P = 1.56 × 10^−24^; interaction between face size and ROI type: F(3,678) = 10.9, P = 5.36 × 10^−7^; interaction between ROI type and fixation order: F(9,678) = 6.73, P = 2.86 × 10^−9^; all other Ps > 0.05). For both large and small faces, subjects started from the face center (Fig. 5C; due to the central cross preceding the face) and then looked at the eyes (Fig. 5A) and mouth (Fig. 5B). Therefore, the fixation density decreased for the center ROI (Fig. 5C; comparing fixation 1 to fixation 2–4: two-tailed paired t-test: large: all Ps < 0.001; small: all Ps < 0.05), and increased for the eye ROI (Fig. 5A; large: Ps < 0.05 between fixation 1 and fixation 2, 4; small: P < 0.05 between fixation 1 and fixation 4) and mouth ROI (Fig. 5B; large: Ps < 0.05 between fixation 1 and fixation 2–4; small: P < 0.05 between fixation 1 and fixation 3). Notably, when comparing large vs. small faces, the difference in the eye ROI was not only at a particular fixation but it was across fixations. Similarly, across fixations, there were only a small proportion of fixations outside ROIs for large faces but the proportion was significantly larger for small faces (Fig. 5D), confirming the wider dispersion of fixations for small faces. Together, this temporal analysis of fixations showed that the differences between large and small faces were not restricted to a particular fixation in serial order but was across fixations.

### Fixation density across morph levels, ambiguity levels, and emotion judgments

**Figure 6:**
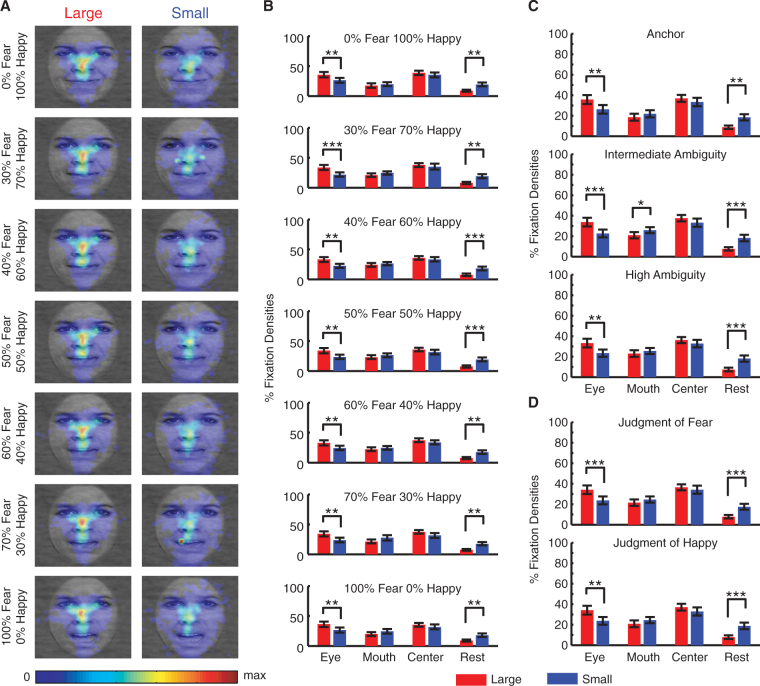
Fixation densities as a function of stimulus level and behavioral judgment. **(A)** Fixation density maps for each morph level. Conventions as Fig. 3B. Face images were constructed from^44^. **(B)** Fixation density for each morph level. **(C)** Fixation density for each ambiguity level. **(D)** Fixation density for each behavioral judgment. Error bars denote ± SEM across subjects. Asterisks indicate significant difference using two-tailed paired t-test. *P < 0.05, **P < 0.01, and ***P < 0.001.

In this last section, I analyzed whether fixation density was modulated by stimulus level and/or emotion judgment. First, for both large and small faces, fixation density maps did not vary as a function of morph levels (Fig. 6A; three-way repeated-measure ANOVA of face size X ROI type X morph level; main effect of ROI type: F(3,1222) = 99.5, P = 1.31 × 10^−57^; interaction between face size and ROI type: F(3,1222) = 22.1, P = 6.07 × 10^−14^; all other Ps > 0.05). Direct comparisons between large and small faces in each ROI (Fig. 6B) showed a significant difference in the eyes (all Ps < 0.005) but not in the mouth or center, with large faces having more fixations onto the eyes and small faces having more fixations outside ROIs. Furthermore, for both large and small faces, fixation density maps did not vary as a function of ambiguity levels (Fig. 6C; three-way repeated-measure ANOVA of face size X ROI type X ambiguity level; main effect of ROI type: F(3,498) = 43.1, P = 9.25 × 10^−25^; interaction between face size and ROI type: F(3,498) = 9.52, P = 4.02 × 10^−6^; all other Ps > 0.05).

I next analyzed fixation densities separately for each behavioral judgment (Fig. 6D; three-way repeated-measure ANOVA of face size X ROI type X judgment; main effect of ROI type: F(3,318) = 28.4, P = 2.59 × 10^−16^; interaction between face size and ROI type: F(3,318) = 6.14, P = 4.51 × 10^−4^; all other Ps > 0.05). Again, similar patterns of fixation densities were found and large faces attracted more fixations to the eyes.

Together, I found that more fixations were directed to the eyes when people viewed larger faces whereas there was a wider spatial dispersion when people viewed smaller faces. However, this difference was independent of the morph level, ambiguity level, and behavioral judgment.

## Discussion

In this study, I employed an emotion judgment task to study whether a difference in stimulus size could influence emotion judgment. I also conducted concurrent eye tracking to reveal the underlying mechanism. I found that face size influenced the threshold to report an emotion as well as specificity in emotion judgment, but not confidence judgment. Eye movement data showed that larger faces attracted more fixations to the eyes whereas smaller faces featured a wider spatial dispersion of fixations. This difference was present across fixations in serial order, and was present at all morph levels, ambiguity levels, and behavioral judgments. Therefore, I demonstrated that face size could bias emotion judgment through eye movement.

I found that large faces resulted in increased judgment of fear and attracted more fixations towards the eyes. Consistent with this finding, it has been shown that the eye region attracts more fixations when people view fearful faces whereas the mouth region attracts more fixations when people view happy faces^19^. Also, eyes contain more information for fearful faces but the mouth contains more information for happy faces^20^. The present study not only revealed an impact of stimulus size on emotion judgment, but also suggested a link between emotion judgment and eye movement, which might explain the underlying mechanism for such modulation by stimulus size. To explore a more direct relationship between emotion judgment and eye movement, I found that the difference in slope (*α*) was marginally significantly correlated with the difference in fixation density in the eye ROI (r = 0.36, P = 0.088), indicating that the more fixations onto eyes, the more sensitive the emotion judgment. However, this curious relationship was still primitive and limited by statistical power. Future studies with a larger sample size and/or a more sensitive task will be needed to show a direct relationship between emotion judgment and eye movement under the modulation of stimulus size.

It is worth noting that visual acuity was normal or corrected-to-normal for all subjects, and the small faces were still large enough (5.95° × 5.95°) so that the details of the faces were clearly visible to subjects. Therefore, the difference in emotion judgment and eye movement between large and small faces was not likely due to differences in low-level stimulus features and visual processing. This was further supported by comparable RTs and confidence ratings between face sizes (Fig. 2). Furthermore, fewer fixations attracted to the eye region for small faces might be due to that when viewing small faces, the eye region was within the foveal or parafoveal area so that upward saccades were not necessary. However, foveation could not fully explain my result, because (1) in contrary to the idea that fewer saccades were needed to sample facial ROIs, small faces did not have more fixations in the image center (note that faces were preceded by a central fixation) but had more distributed fixations compared to large faces (Fig. 3 and Fig. 4A), even outside the ROIs (Fig. 4A), suggesting that subjects still made saccades (including upward saccades) to sample the faces. Notably, subjects had comparable fixations onto the mouth (Fig. 4A), again showing that subjects still saccaded to facial ROIs when viewing small faces. (2) Importantly, large faces had asymmetric fixation densities along the vertical axis, with more fixations directed upwards to the eyes (34.1 ± 20.8% (mean ± SD)) but fewer downwards to the mouth (21.2 ± 15.7%), whereas small faces had a rather symmetric vertical distribution, with similar fixation densities for the eyes (23.3 ± 18.5%) and mouth (24.7 ± 14.3%; Fig. 3 and Fig. 4A). The difference in fixation density between the eyes and mouth further confirmed this finding (large: 12.9 ± 34.8%, small: −1.47 ± 30.0%; two-tailed paired t-test: t(23) = 3.15, P = 0.0045, g = 0.44). Therefore, the difference in fixations onto eyes between small and large faces was primarily due to selective upward saccades when viewing large faces, a psychological process probably related to distance (see below), rather than a simple consequence of foveation. Notably, such upward saccades towards the eyes can be in turn attributed to amygdala activation^21^.

Stimulus size is an important factor in optical^26,27^, visual arts^28^, and psychophysiological^29,30^ research. In one very early study, it has been showed that, in general, medium size circles are preferred over either larger or smaller circles^31^. Later studies using art works showed that the size-distance relationship influences judgments of preference and interestingness as well as the duration of looking time^28^. Stimulus size directly affects retinal projection and it is in turn affected by viewing distance. However, it has been shown that perceived distance and stimulus size are judged quite independently from the retinal and extra-retinal information, in the sense that no attempt is made to attain consistency^32^. Psychological studies have shown that stimulus size influences motion aftereffect^33^ and affects attention^34^. Physiologically, the polarity and saturation profile of the visual evoked potential change as a function of stimulus size^30^. Furthermore, the amplitude of the visual gamma-band response is diminished for small and peripheral stimuli^35^. In particular, although significant emotional modulation of event-related potentials is observed across stimulus sizes at both earlier and later stages of processing, the modulation of earlier processes is reduced in smaller compared to larger stimulus sizes^36^. Consistent with this result, when viewers respond to video images from television and film that display different emotions, the largest stimulus produces greater heart rate deceleration as well as greater skin conductance than the medium and small stimuli, suggesting a more pronounced emotional response for larger stimuli^37^.

Larger stimuli indicate a smaller distance between the observer and the encountered stimuli. Therefore, a plausible explanation of the present result is that larger faces indicate a smaller interpersonal distance. Consistent with the lower threshold to report fear with larger faces in the present study, it has been shown that perspective distortion from interpersonal distance is an implicit visual cue for social judgments of faces: photographs of faces taken from within personal space elicit lower investments in economic trust game and lower ratings of social traits (such as trustworthiness, competence, and attractiveness), compared to photographs taken from a greater distance^38^. On the other hand, the lower threshold to report fear with larger faces might indicate that for a given level of fear-happy morph, subjects perceived more fearful emotion with large faces, consistent with larger emotional responses elicited by larger (thus indicated nearer) stimuli^36^. Relatedly, snake-phobic people show a linear increase of autonomic responses and self-reported fear as a function of distance to snakes^39^. Because distance and retinal size are strictly related^40^, it can be expected that changes in stimulus size determine arousal modulations similarly to distance. This is thus consistent with the increased judgment of fear with larger faces in the present study.

Using movie shots, it has been shown that smaller faces take longer to categorize the valence of facial expressions than those that are larger, and more clutter creates crowding and impedes the interpretation of expressions for more distant faces but not proximal ones^41^. This indicates an attentional mechanism underlying emotion judgment related to stimulus size, consistent with the result from computational modeling showing that object-level saliency (including face size) contributes to attract more fixations to faces^5^. Neural processing of facial expressions of emotions appears to require attention^42^; and therefore another plausible explanation of the size effect might be that different stimulus sizes elicited different attention. Although in the present study I found that RT was comparable between large and small faces (Fig. 2), this might be due to that my subjects were only allowed to respond after the offset of the stimulus, therefore, they might make their decisions well before executing the button press. The task was not fully speeded because it was designed to better dissociate perception and decision; however, a fully speeded version of the task can well replicate the emotion judgment result^43^.

In the past decades, there have been numerous studies investigating facial expressions of emotions. However, most studies investigating emotion judgment focus on the emotion contents as well as the facial features related to emotions, but rarely investigate whether a simple low-level feature, face size, can affect emotion judgment. In this study, I revealed not only a difference in emotion judgment, but also a possible attribution to eye movements. The present result implies that future studies investigating emotion judgment should consider stimulus size carefully, especially for comparisons between different subject groups and investigations across different research sites. A future direction is to test whether the present result can be extended to other facial emotions and more complex social traits such as trustworthiness, dominance, and guilt. It also remains to investigate the neural mechanisms underlying the modulation by stimulus size.

## Methods

### Subjects

There were 24 subjects (16 female, 22.3 ± 3.39 years). Subjects gave written informed consent according to protocols approved by the institutional review board of the South China Normal University, and all methods were carried out in accordance with the approved guidelines. Visual acuity was normal or corrected-to-normal for all subjects. One subject was excluded from emotion judgment analysis because the psychometric function could not be fitted.

### Task and stimuli

I used an established task and stimuli to study emotion judgment^15,43–45^. Subjects viewed a face for 1 second and were asked to report their judgment of the facial emotion (fearful or happy) as quickly as possible (Fig. 1A). Subjects had to respond within 2 seconds after stimulus offset. Following emotion judgment, subjects were also asked to report their confidence in their emotion judgment (3 levels: very sure, sure, and unsure). There was no correct answer to the emotion judgment or confidence judgment (purely subjective), and no feedback was thus provided to subjects. After confidence judgment, a central cross was displayed for 1 to 2 seconds before the next trial started.

Morphed faces were created from anchor faces with unambiguous fearful or happy expressions. There were 4 face models (2 female) and 5 levels of morphs: 30% fear/70% happy, 40% fear/60% happy, 50% fear/50% happy, 60% fear/40% happy, and 70% fear/30% happy (Fig. 1B). All stimuli had equal low-level image properties^15^.

Two face sizes were tested in this study. Large faces subtended a visual angle of 11.9° × 11.9°, and small faces subtended a visual angle of 5.95° × 5.95°. There were 252 trials in 3 consecutive blocks (36 trials per morph level) for large faces, and 252 trials in 3 consecutive blocks for small faces. The order of blocks of large faces and small faces was counterbalanced. I also ensured that there was no order, adaptation, or practice effect: *x*_*half*_ was similar for large faces preceding vs. following small faces (preceding: 47.1 ± 5.00 (mean ± SD), following: 49.3 ± 5.15; two-tailed unpaired t-test: t(21) = 1.06, P = 0.30, g = 0.43) and it was also the case for small faces (preceding: 51.2 ± 6.00, following: 51.4 ± 6.08; t(21) = 0.051, P = 0.96, g = 0.021). Similarly, *α* was similar for large faces preceding vs. following small faces (preceding: 0.14 ± 0.064, following: 0.15 ± 0.061; t(21) = 0.32, P = 0.76, g = 0.13) and it was also the case for small faces (preceding: 0.11 ± 0.038, following: 0.13 ± 0.052; t(21) = 0.85, P = 0.41, g = 0.34).

### Psychometric curve

I used a logistic function to fit smooth psychometric curves (Fig. 1C):

1 $$P(x)=\frac{{P}_{\inf }}{1+{e}^{-\alpha (x-{x}_{half})}}$$

where *x* is the stimulus level, *P* is the proportion of trials of fearful judgment, *P*_*inf*_ is the curve’s maximum value when *x* approaches infinity, *x*_*half*_ is the curve’s midpoint (i.e., symmetric inflection point), and *α* is the slope (steepness) of the curve. The parameters *P*_*inf*_, *x*_*half*_, and *α* were derived from the observed data (*P* and *x*) for each subject, and *x*_*half*_ and *α* were used to compare emotion judgment. Specifically, *x*_*half*_ shows emotion judgment bias and *α* shows emotion judgment sensitivity and specificity.

### Eye tracking

Two eye trackers were used in this study. Fourteen subjects were recorded using an EyeLink 1000 System (SR Research, Canada) and ten subjects were recorded using a Tobii T120 system. EyeLink tracked one of the eyes at 1000 Hz and Tobii tracked both eyes at 120 Hz. In both experiment setups, MATLAB with the Psychophysics Toolbox^46^ was used and the viewing distance was approximately 60 cm. Calibration was performed at the beginning of each block. In experiments with EyeLink, fixations and saccades were extracted using the software supplied with EyeLink (deflection threshold = 0.1°, velocity threshold = 30°/s, and acceleration threshold = 8000°/s^2^). In experiments with Tobii, fixations and saccades were extracted using Tobii Fixation Filter^47^ implemented in Tobii Studio (velocity threshold = 35 [pixels/samples] and distance threshold = 35 [pixels]).

Rectangular ROIs were drawn to encompass 3 facial features: eyes, mouth, and center (Fig. 3B; note that eye ROI has the same size as mouth ROI). Fixation density maps were derived by smoothing fixation locations using a 2D Gaussian kernel (size = 40 pixels, SD = 10 pixels) and were then normalized within each subject. Fixation density maps represent the likelihood of fixating a particular location of the stimulus and are shown in arbitrary units.

### Statistics

Because each subject viewed both large faces and small faces, two-tailed paired t-tests and repeated-measure ANOVAs were used throughout the analyses. Specifically, paired t-tests were used to compare the indices of emotion judgment (Fig. 1), and one-way or two-way repeated-measure ANOVAs were used to compare confidence judgments (Fig. 2). To compare the spatial distribution of fixations (Fig. 3A), point-by-point two-tailed paired t-test was used and further corrected by false discovery rate (FDR)^48^. To compare fixation density maps (Fig. 3D), pixel-by-pixel two-tailed paired t-test was used, but it was uncorrected for multiple comparisons. In all ROI analyses (Figs 4–6 **)**, ANOVAs with multiple factors were first performed, followed by post-hoc two-tailed paired t-tests between face sizes for each ROI to further confirm the findings. MATLAB was used to compute statistics. Detailed statistical measures were also specified before each result.
